# Laser Printing of Superhydrophobic Patterns from Mixtures of Hydrophobic Silica Nanoparticles and Toner Powder

**DOI:** 10.1038/srep36735

**Published:** 2016-11-08

**Authors:** Chi-Vinh Ngo, Doo-Man Chun

**Affiliations:** 1 School of Mechanical Engineering, University of Ulsan, Ulsan, South Korea

## Abstract

In this work, a new and facile dry printing method was developed for the direct fabrication of superhydrophobic patterns based on silica nanoparticles. Mixtures of hydrophobic fumed silica nanoparticles and toner powder were printed on paper and polymer sheets using a commercial laser printer to produce the superhydrophobic patterns. The mixing ratio of the toner powder (for the laser printer) to hydrophobic silica was also investigated to optimize both the printing quality and the superhydrophobicity of the printed areas. The proper mixing ratio was then used to print various superhydrophobic patterns, including triangular, square, circular, and complex arrangements, to demonstrate that superhydrophobic surfaces with different patterns can be fabricated in a few seconds without any post-processing. The superhydrophobicity of each sample was evaluated by contact angle measurements, and all printed areas showed contact angles greater than 150°. The research described here opens the possibility of rapid production of superhydrophobic surfaces with various patterns. Ultimately, the obtained findings may have a significant impact on applications related to self-cleaning, control of water geometry and position, fluid mixing and fluid transport.

The wettability of a solid surface by water, i.e., the degree to which a surface may be classified as superhydrophilic, hydrophilic, hydrophobic, or superhydrophobic, is determined by two factors: the surface energy and the surface structure. Micro/nano or hierarchical structures (often described as rough structures) of low-surface-energy materials typically display increased hydrophobicity. Consequently, such structures may form surfaces with significant anti-wetting (superhydrophobic) characteristics. A superhydrophobic surface is defined as a surface with a water droplet contact angle (WDCA) greater than 150° and a sliding angle (SA) less than 10°. Many technologies have been developed for the fabrication of superhydrophobic surfaces. Two types of strategies have been employed to produce superhydrophobic surfaces: (1) forming a rough surface from a low-surface-energy material^1^,^2^,^3^,^4^,^5^,^6^,^7^,^8^,^9^,^10^,^11^ and (2) modifying a surface using a low-surface-energy material^12^,^13^,^14^,^15^,^16^,^17^,^18^,^19^,^20^,^21^.

Recently, the important role of patterned superhydrophobic surfaces was demonstrated in water collection^22^,^23^,^24^,^25^, and patterned superhydrophobic surfaces have been used in various applications, such as self-cleaning, anti-fogging, open-air microchannel devices, and lab-on-chip devices^26^,^27^,^28^,^29^,^30^,^31^. For the facile fabrication of patterns on a surface, inkjet, wax, and laser printing procedures are commonly used. Although inkjet printing has previously been employed at various stages for the production of superhydrophobic patterns^29^,^31^,^32^,^33^,^34^,^35^,^36^,^37^,^38^,^39^,^40^,^41^,^42^,^43^, the direct fabrication of superhydrophobic patterns by inkjet printing has not yet been achieved. In the studies using inkjet printing, a superhydrophobic surface was first produced by other means, such as chemical vapor deposition (CVD), spray coating, or dip coating^22^,^30^,^31^,^44^,^45^. A hydrophilic/superhydrophilic surface was then inkjet-printed with the prepared materials to fabricate the hydrophilic/superhydrophilic-superhydrophobic patterns. In another study, Barona and Amirfazli investigated the wetting characteristics of superhydrophobic paper by inkjet and laser printing^45^. However, much of their work focused on the effects of the hydrophilic ink intensity on the prepared superhydrophobic paper.

In this work, we developed a simple approach for the preparation of dry powder mixtures of hydrophobic silica nanoparticles and toner powders to facilitate the direct production of superhydrophobic surfaces with a commercial laser printer. Hydrophobic fumed silica nanoparticles were mixed mechanically with commercial toner powders and were subsequently printed on paper and polymer sheets using a commercial laser printer. The mixing ratio of the toner powder to hydrophobic silica nanoparticles was varied, and the proportion required to obtain the optimal printing quality and superhydrophobicity was determined. Various superhydrophobic patterns, including triangular, square, and circular arrangements, were ultimately printed on paper and polymer sheets using a laser printer to demonstrate that superhydrophobic surfaces with complex configurations could be fabricated in a few seconds without any additional post-processing, such as drying or heat treatment. The devised synthesis method could allow the production of various superhydrophobic patterns rapidly in a home, office, or laboratory. Furthermore, the strategy described here may find extensive use in many potential applications related to microfluidic devices, fluid transportation, self-cleaning, and control of water geometry and position.

## Results and Discussion

### Water droplet contact angle (WDCA)

**Figure 1:**
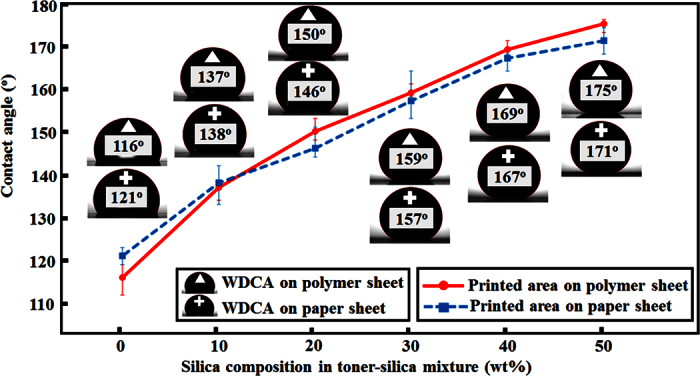
Contact angles measured for polymer and paper sheets with the properly printed areas of different silica compositions.

**Figure 2:**
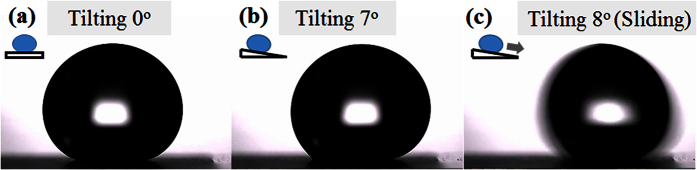
Sliding angle of the properly printed area on polymer sheet with 30 wt% of silica nanoparticles in the toner-silica mixture. (**a**) 0° tilting angle, (**b**) 7° tilting angle, and (**c**) 8° tilting angle.

The wettability of samples with different mixing ratios of silica from 0 wt% to 50 wt% was evaluated. In the absence of printing, the bare polymer sheet showed hydrophilic (average WDCA of 70°) properties, whereas the bare paper sheet exhibited hydrophobic (average WDCA of 124°) characteristics. The measured water contact angles changed after printing the toner-silica mixtures. As shown in Fig. 1, the contact angle on the areas properly printed on paper and polymer sheets increased as the weight percent of fumed silica nanoparticles (from 0 to 50 wt%) was raised. The contact angles on the areas properly printed on both the paper and polymer sheets showed contact angle values greater than 150° when the fraction of silica in the toner-silica mixture was 30 wt% or higher. To characterize whether the influence of a sessile water droplet’s weight on the contact angle could be ignored, the Bond number (Bo) was calculated. The calculated Bond numbers of the samples printed with the silica composition of 30 wt% in the toner-silica mixture on the polymer and paper were 0.09 and 0.078, respectively, with these values becoming smaller as the silica composition increased to higher than 30 wt%. All Bond numbers were much smaller than 1. Therefore, the influence of the sessile water droplet’s weight on the contact angle with the volume of water droplet was not dominant. To confirm the superhydrophobic property, the sliding angle was measured on the sample made with 30 wt% of silica nanoparticles in the toner-silica mixture. A water droplet (11 μL) was placed on the printed area of the polymer without tilting, as shown in Fig. 2. Then, the surface was tilted slowly (tilting speed: 0.15 °/s). The water droplet started sliding at an 8° tilting angle. On the paper substrate, the sliding angle could not be measured properly owing to the water absorption of the paper and the deformation of the paper sheet during the measurement. Overall, the specimens exhibited superhydrophobic properties at silica fractions of 30 wt% or higher and hydrophobic characteristics at silica ratios lower than 30 wt%. In addition, the wetting state of the initially hydrophilic polymer sheet was changed to hydrophobic or superhydrophobic in the presence of the toner or toner-silica mixtures. This finding can be used to induce different levels of wettability on polymer films.

### Printing quality

**Figure 3:**
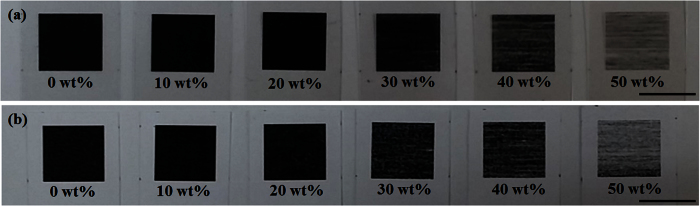
Printing quality of toner-silica mixtures with different fractions of silica nanoparticles on (**a**) polymer sheets and (**b**) paper; the scale bar is 10 mm.

The printing quality on the paper and polymer sheet decreased with an increasing fraction of silica nanoparticles, as shown in Fig. 3. In the toner-silica mixtures with silica fractions lower than 30 wt%, the printed areas (squares) were almost black. Several white regions or white lines combined with dark areas were observed, especially for the samples with 40 and 50 wt% silica. The white color represented the color of the bare paper substrate or white background material for transparent polymer substrate. The white regions or lines produced using 40 and 50 wt% silica, as shown in Fig. 3, were not printed properly with the toner-silica mixtures owing to the presence of a large amount of the hydrophobic silica nanoparticles.

### Morphology

**Figure 4:**
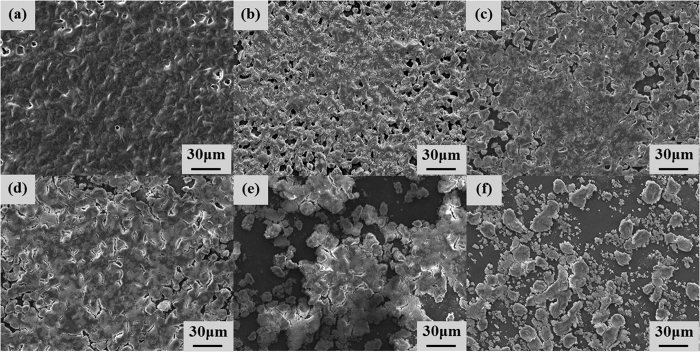
FESEM images of samples properly printed on polymer sheets with different toner-silica ratios. (**a**) 100-0 wt%, (**b**) 90-10 wt%, (**c**) 80-20 wt%, (**d**) 70-30 wt%, (**e**) 60-40 wt%, and (**f**) 50-50 wt%.

**Figure 5:**
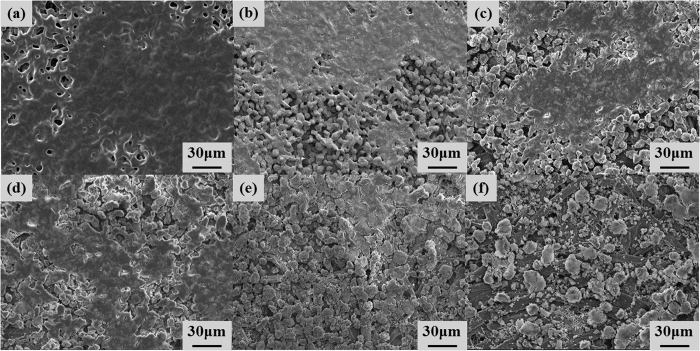
FESEM images of samples properly printed on paper with different toner-silica ratios. (**a**) 100-0 wt%, (**b**) 90-10 wt%, (**c**) 80-20 wt%, (**d**) 70-30 wt%, (**e**) 60-40 wt%, and (**f**) 50-50 wt%.

**Figure 6:**
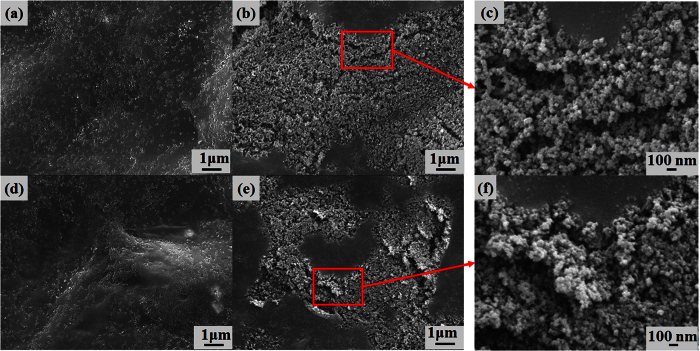
FESEM images obtained for fabricated samples. (**a**) 100-0 wt% toner-silica on a polymer sheet, (**b**) 70-30 wt% toner-silica on a polymer sheet, (**c**) enlarged image of (**b**), (**d**) 100-0 wt% toner-silica on paper, (**e**) 70-30 wt% toner-silica on paper, and (**f**) enlarged image of (**e**).

The surfaces of the samples were examined by field emission scanning electron microscopy (FESEM, JSM-6500F, Jeol Co., Japan); the microstructures on the polymer and paper were observed as shown in Figs 4 and 5, respectively. After printing with 100 wt% toner, several regions that were not covered by the powders as pits were observed on the paper, whereas very small regions not covered by the powders were observed on the polymer. When printing with toner-silica mixtures on the surfaces, the regions that were not covered by the powders gradually increased. However, the deformed particles bonded together could still cover most of the surface area, as shown in Figs 4(a–d) and 5(a–d). The samples showed almost black, as shown in Fig. 3. The printing quality on the polymer sheet was better than that on the paper sheet with the same ratio as that for the toner-silica mixtures. When the silica fraction was increased to above 30 wt%, the particles could not cover most of the surface area and the covered regions became separated on the paper and polymer sheets; this phenomenon made the color change from black to gray, as shown in Fig. 3. This finding was attributed to the hydrophobic nature of the silica nanoparticles, which can block the deposition of the toner powder on the substrate and bond to other toner particles. A sufficient toner ratio is necessary for proper printing, and the toner served as a binder between the silica nanoparticles and the substrate. In addition to the microstructures, nanostructures could be observed only on the samples made with toner-silica mixture, as shown in Fig. 6. The samples printed with 100 wt% toner showed smooth surface in nanoscale, whereas the samples printed with the toner-silica mixture showed nanostructures on the top of surface.

### Mechanism

**Figure 7:**
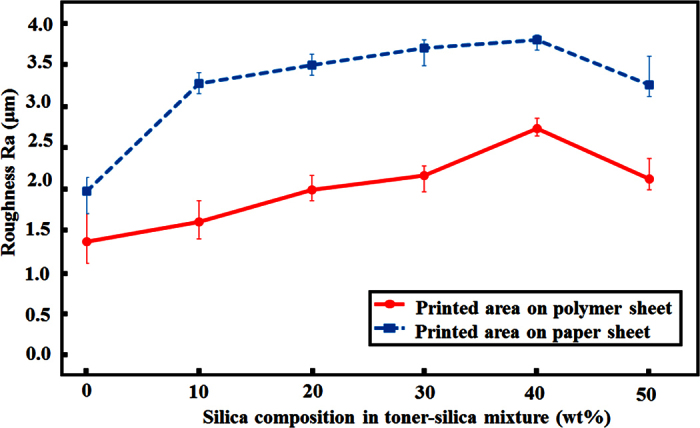
Surface roughness (Ra) measured for polymer and paper sheets with the properly printed areas of different silica compositions.

Superhydrophobicity can be caused by two factors: a low surface energy and a rough surface that includes microstructures and nanostructures. A rough surface can give rise to the partial wetting conditions by trapping the air between the water droplet and solid surface and a reduction of the water contact area to solid surface. A low surface energy could be achieved by the hydrophobic silica nanoparticle. When the fraction of the hydrophobic silica nanoparticles in the toner-silica mixture was increased, the amount of silica on the top surface also increased, and the surface energy of the printed surface decreased. The surface roughness (Ra) of the samples at the microscale was measured by confocal microscopy 3D and profile measurement equipment (VK-X200 series, Keyence, Japan). The polymer was hydrophilic, with a low surface roughness of 0.1 μm, whereas the paper sheet was hydrophobic, with a high surface roughness of 3.2 μm. The initial properties could affect the bonding between the toner powder during laser printing. As shown in Fig. 7, the addition of more silica nanoparticles increased the surface roughness, whereas the surface roughness of the samples with 50 wt% silica nanoparticles in the toner-silica mixture decreased because of the appearance of many regions that were not covered by the powder. However, the water droplet contact angle increased on the properly printed area even with 50 wt% silica because the rough structure may generate the partial wetting conditions. In addition to microstructures, nanostructures could be observed only on the samples made with the toner-silica mixture, as shown in Fig. 6. The nano/micro hierarchical structures on the printed areas could enhance the contact angle because they can generate the partial wetting condition by trapping the air with the nanostructure and the microstructure. Therefore, nano/micro hierarchical structures with low surface energies were formed with a sufficient silica composition in the toner-silica mixture and were able to make the printed area superhydrophobic.

**Figure 8:**
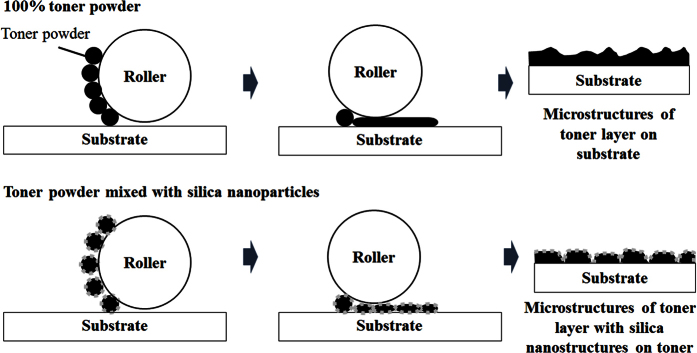
Mechanism of fabricating patterned superhydrophobic surfaces by laser printing.

The mechanism of this phenomenon is illustrated simply in Fig. 8. When only the toner was printed on the paper or polymer sheets, the samples were hydrophobic. However, the printing of the toner on the paper or polymer sheets with a silica fraction of 30 wt% or higher yielded superhydrophobic specimens. In the case of the pure toner powder, the roller would deform the toner by pressure and heat. Consequently, the toner powder was printed on the substrate and formed a thin film with distinct microstructures. The contact angle changed owing to the existence of the microstructures and the inherently low surface energy of the toner powder such that the resulting surface was hydrophobic. Similarly, the roller deformed mixtures of the toner and silica powder by pressure and heat during the printing of the patterns on the substrate. Here, the silica nanoparticles can stick to the deformed toner powder, and the toner powder can also adhere to the substrate. A superhydrophobic nano/micro hierarchical structure with a low surface energy was thus produced owing to the incorporation of silica nanoparticles. However, an undesirably large amount of silica nanoparticles hindered the bonding of the toner to the substrate, which led to a reduction in both the powder-covered regions and the printing quality at silica fractions of 40 wt% or higher. Especially, the samples with 50 wt% of silica showed relatively clear white regions or lines, which were not printed properly, and the contact angle on this region was lower than 150°. Therefore, the samples with 30 wt% of the hydrophobic silica nanoparticles in the toner-mixture showed a good combination of superhydrophobicity and acceptable printing quality.

### Various printable superhydrophobic patterns

**Figure 9:**
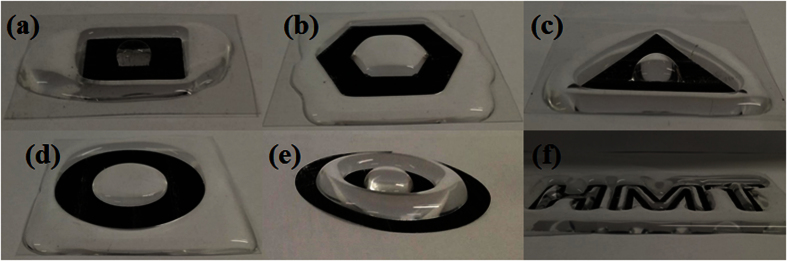
Various superhydrophobic patterns fabricated on polymer sheets. (**a**) square frame shape, (**b**) hexagonal frame shape, (**c**) triangular frame shape, (**d**) circular frame shape, (**e**) double-circular frame shape, and (**f**) letters.

Simple and complex patterns directly printed from the mixtures with the optimal 30 wt% fraction of the silica on the polymer sheets are presented in Fig. 9. The fabrication time was the same as that associated with commercial laser printing (depending on the speed of the laser printer), and all of the printed patterns were superhydrophobic. Water droplets easily slid off the superhydrophobic areas (black regions) and were subsequently maintained in the regions that were not superhydrophobic (white/transparent areas or bare substrates). (Supplementary Video S1).

**Figure 10:**
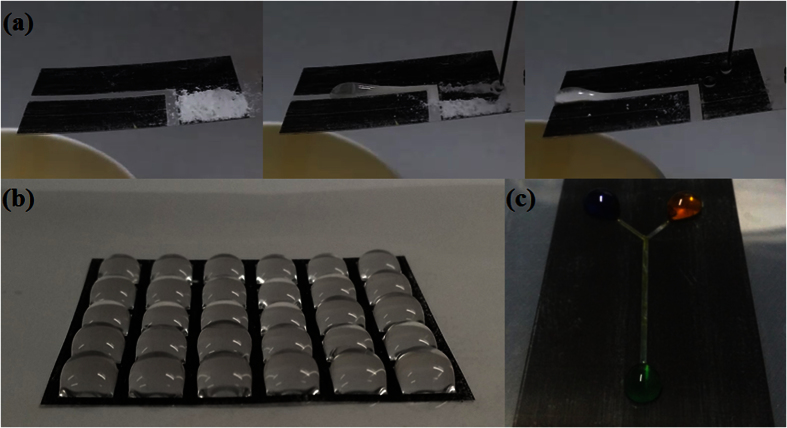
Potential application of laser printed superhydrophobic patterns on polymer. (**a**) self-cleaning, (**b**) water droplet positioning, and (**c**) water droplet mixing.

Potential applications such as self-cleaning, liquid droplet positioning, and open-air channel can be suggested using this simple printing technique with proper pattern designs. The self-cleaning was introduced in Fig. 10(a) and Supplementary Video S2. Water droplets could clean the white dusts on the printed superhydrophobic area with tilting angle of 8°. The sliding water droplets took the white dust powders from the printed area and slid along the non-printed track pattern. The liquid droplet positioning for liquid micro-array was demonstrated with superhydrophobic multi-square patterns on a hydrophilic polymer substrate, as shown in Fig. 10(b). Using the designed patterns, water was controlled in different geometries and positions. Further, open-air channels for liquid mixing are shown in Fig. 10(c). By using proper open-air channels, a fluid could be transported in an oriented track, and two fluids could be mixed together. (Supplementary Video S3).

## Conclusions

In conclusion, various superhydrophobic patterns could be directly fabricated by a laser printer in a few seconds using a simple method without any additional post-processing. Moreover, a mixing ratio of toner powder to silica nanoparticles (70 wt% toner and 30 wt% silica) was determined for the optimal combination of printing quality and superhydrophobicity. The mechanisms of superhydrophobicity and the synthesis process were also discussed. The devised laser printing technique for direct superhydrophobic patterning has the potential to enable many possible applications, such as self-cleaning, control of water geometry and position, fluid mixing and fluid transport. Furthermore, because most homes, offices, and laboratories have a laser printer, the technique described in this work can be easily implemented in a variety of settings.

## Methods

### Material preparation

Superhydrophobic patterns were fabricated using a commercial laser printer (SCX-3205W, Samsung, Korea) with toner-silica mixtures placed into the toner cartridge. Hydrophobic fumed silica nanoparticles (Primary particle size of 7–40 nm, SiO_2_, Konasil, K-P20, OCI Co. Ltd., Korea) were used for the printing material. The toner powder (CLT-K407S, Samsung, Korea) was used as a binder between the silica and the substrates. The toner size was about 11 μm and the toner composition included styrene acrylic resin (75~85%), wax (5~10%), cyan pigment (1~6%) and silica (1~3%)^46^. Commercial printing paper (A4, 210 × 297 mm, Double A, Korea) and polymer sheets (Polyester, PP3300, Great, Korea) were used as the substrates.

### Pattern fabrication

**Figure 11:**
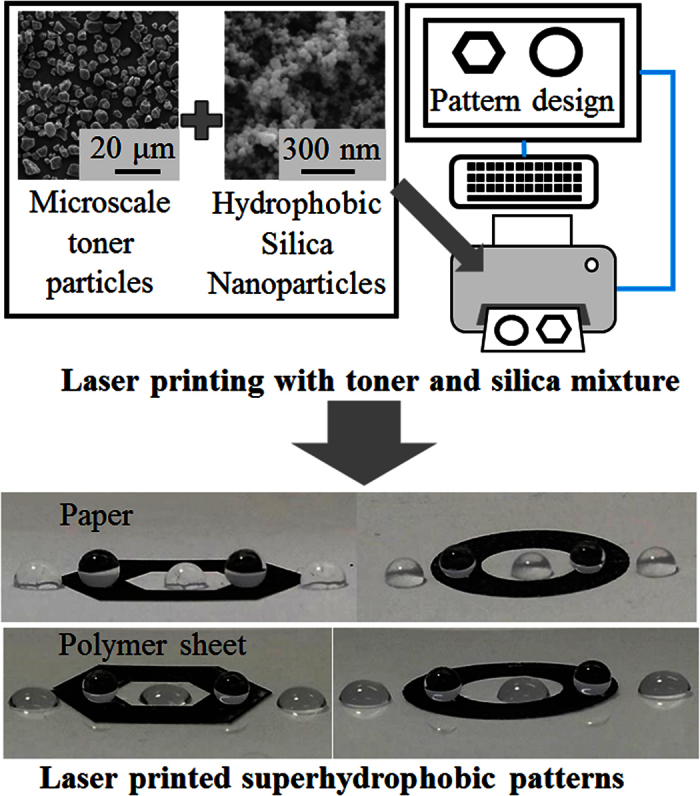
Process of fabricating superhydrophobic patterns by laser printing.

To obtain sufficient printing quality and superhydrophobicity, a series of samples with 0 to 50 wt% silica in the toner-silica mixture were prepared; the ratio of silica was increased by 10 wt% for each sample set. Each toner-silica composition was prepared five times. The procedure for fabricating the patterns is summarized in Fig. 11. Mixtures of the as-purchased toner powder and hydrophobic silica nanoparticles were ultimately prepared by ball milling at 150 rpm for 15 min. The mixtures were placed into the material chamber of the laser printer toner cartridges. The toner cartridges were then inserted into the laser printer. Patterns were designed using the widely used Microsoft Powerpoint 2010 software, and the toner-silica mixtures were printed on paper and polymer sheets by the laser printer. Finally, contact angle measurements using a sessile water droplet with a volume of 11 μL were carried out to determine the wetting state of the samples by a contact angle meter (SmartDrop, Femtofab Co. Ltd., Korea). For each specimen, the contact angle was taken as the average of three measurements. The average, maximum, and minimum contact angle values at each toner-silica ratio were calculated from the average contact angle values of the five samples prepared at identical silica contents. A proper ratio of toner to silica was chosen after contact angle measurements and a comparison of printing quality.

## Additional Information

**How to cite this article**: Ngo, C.-V. and Chun, D.-M. Laser Printing of Superhydrophobic Patterns from Mixtures of Hydrophobic Silica Nanoparticles and Toner Powder. *Sci. Rep.* **6**, 36735; doi: 10.1038/srep36735 (2016).

**Publisher’s note:** Springer Nature remains neutral with regard to jurisdictional claims in published maps and institutional affiliations.

## Supplementary Material

Supplementary Information

Supplementary Information

Supplementary Information

Supplementary Information
