# Supplementary material for: Laser Printing of Superhydrophobic Patterns from Mixtures of Hydrophobic Silica Nanoparticles and Toner Powder
Source: Sci Rep. 2016 Nov 8;6:36735. doi: 10.1038/srep36735 (PMC5099995; doi:10.1038/srep36735)
Supplement: Supplementary Information [file srep36735-s1.pdf]

Laser Printing of Superhydrophobic Patterns from Mixtures of Hydrophobic Silica Nanoparticles and Toner Powder

Chi-Vinh Ngo[1], and Doo-Man Chun[1,*]

[1]School of Mechanical Engineering, University of Ulsan, Ulsan, South Korea.

[*]Corresponding author's email: dmchun@ulsan.ac.kr

Telephone: +82-52-259-2706

SUPPLEMENTARY INFORMATION

Video legends:

Video S1 shows that the fabrication time to make the superhydrophobic patterns was a few seconds, depending on the speed of the laser printer. The designed patterns were easily printed as a conventional laser printing. Furthermore, the water could be positioned by the printed patterns.

Video S2 shows potential application of laser printed superhydrophobic patterns on polymer as self-cleaning. Water droplets could clean the white dusts on the printed superhydrophobic area with tilting angle of 8°. The sliding water droplets took the white dust powders from the printed area and slid along the non-printed track pattern.

Video S3 shows potential application of laser printed superhydrophobic patterns on polymer as water droplet mixing. A fluid could be transport in an oriented track. Moreover, two fluids could be mixed together.
